# COMPARISON OF HEALTH OUTCOMES AMONG TWO SEPTUAGENARIAN COHORTS A DECADE APART IN JAPAN

**DOI:** 10.1093/geroni/igae098.4144

**Published:** 2024-12-31

**Authors:** Can Wang, Yasuyuki Gondo, Takeshi Nakagawa, Yukie Masui, Kei Kamide, Ayaka Kasuga, Saori Yasumoto, Madoka Ogawa

**Affiliations:** Osaka University, Suita, Osaka, Japan; Osaka University, Suita, Osaka, Japan; Osaka University, Suita, Osaka, Japan; Tokyo Metropolitan Institute of Gerontology, Tokyo, Japan; Osaka University, Suita, Osaka, Japan; Ritsumeikan University, Osaka, Japan; Osaka University, Suita, Osaka, Japan; Osaka University, Suita, Osaka, Japan

## Abstract

**Background:**

Previous studies have suggested that the health status of older adults living in the community has improved significantly over the past two decades. To reassess whether this trend continues, this study examined the health profiles of a current cohort of 70-year-olds and compared them with a cohort born a decade earlier.

**Method:**

This cohort comparison study was part of the observational Septuagenarians, Octogenarians, Nonagenarians Investigation with Centenarians (SONIC) study. Data were collected from two groups of 69 to 71-year-old community-dwelling older adults in 2010 (born between 1938 and 1941 (n=483)) and in 2023(born between 1951 and 1954 (n=283)), using identical methodologies. All participants underwent comprehensive physical, mental, social, and cognitive assessments.

**Result:**

Overall, the later cohort was healthier than the earlier cohort. In physical performance, the later cohort showed 21.08% higher grip strength, 13.65% higher gait speed, 7.02% higher chair-seated stepping, and in cognitive function, the later cohort showed 7.93% higher MOCA-J score. The later cohort also reported higher levels of social support(η2=0.013) and showed higher perceived health(η2=0.011). However, mental health assessed by WHO-5 was lower in later cohort(η2=0.008).

**Conclusion:**

Our findings regarding functional capacity were in-keeping with previous studies, which showed later cohort exhibited superior health outcomes across multiple domains compared to earlier cohort. However, there is an inconsistency with previous studies, which reported that later cohorts had better mental health than earlier cohorts at the same age. Future research should explore the reasons for this discrepancy.

